# Efficientand Robust Automated Segmentation of Nanoparticles and Aggregates from Transmission Electron Microscopy Images with Highly Complex Backgrounds

**DOI:** 10.3390/nano14141169

**Published:** 2024-07-09

**Authors:** Lishi Zhou, Haotian Wen, Inga C. Kuschnerus, Shery L. Y. Chang

**Affiliations:** 1 School of Materials Science and Engineering, University of New South Wales, Sydney, NSW 2052, Australia; lishi.zhou@unswalumni.com (L.Z.); ikuschnerus@ucsd.edu (I.C.K.); 2 Electron Microscope Unit, Mark Wrainwright Analytical Centre, University of New South Wales, Sydney, NSW 2052, Australia

**Keywords:** nanoparticles, image analysis, aggregates, transmission electron microscopy

## Abstract

Morphologies of nanoparticles and aggregates play an important role in their properties for a range of applications. In particular, significant synthesis efforts have been directed toward controlling nanoparticle morphology and aggregation behavior in biomedical applications, as their size and shape have a significant impact on cellular uptake. Among several techniques for morphological characterization, transmission electron microscopy (TEM) can provide direct and accurate characterization of nanoparticle/aggregate morphology details. Nevertheless, manually analyzing a large number of TEM images is still a laborious process. Hence, there has been a surge of interest in employing machine learning methods to analyze nanoparticle size and shape. In order to achieve accurate nanoparticle analysis using machine learning methods, reliable and automated nanoparticle segmentation from TEM images is critical, especially when the nanoparticle image contrast is weak and the background is complex. These challenges are particularly pertinent in biomedical applications. In this work, we demonstrate an efficient, robust, and automated nanoparticle image segmentation method suitable for subsequent machine learning analysis. Our method is robust for noisy, low-electron-dose cryo-TEM images and for TEM cell images with complex, strong-contrast background features. Moreover, our method does not require any a priori training datasets, making it efficient and general. The ability to automatically, reliably, and efficiently segment nanoparticle/aggregate images is critical for advancing precise particle/aggregate control in biomedical applications.

## 1. Introduction

Nanoparticles have a wide range of applications, including biomedical diagnostics [1], energy storage [2], catalysis [3,4], agricultural production [5], and environmental protection [6]. An excellent example is quantum dots, a class of nanoparticles, whose broad impact earned them recognition with the Nobel Chemistry prize in 2023. Among the applications of nanoparticles, biomedical applications [7,8,9,10] such as medical imaging contrast enhancement [11] and drug delivery [12,13] are growing rapidly. The key to the success of these applications is the control of their size, shape, and dispersions. For biomedical applications, the impact of the aforementioned factors on cellular uptake [7,8,9], biomolecule absorption [8], and their cytotoxicity [9,10] is critical in establishing the effectiveness and safety of the synthesized particles [14]. While many methods have been demonstrated to successfully control the size, shape, and dispersions [15,16,17], reliable characterization and analysis methods are still required to validate the nanoparticles to ensure that they meet the desired conditions in their working state.

Transmission electron microscopy (TEM) is a powerful technique capable of providing direct information on the nanoparticle size, shape, and structure down to the atomic level [18,19]. By utilizing TEM imaging under cryogenic conditions, it becomes possible to measure the native state of nanoparticle dispersion by plunge-freezing the dispersion. However, analyzing statistically representative numbers of particles from a large number of images is very laborious. The need for efficiently analyzing large amounts of data has driven the recent development of machine learning (ML) methods [20,21] for automatically identifying and classifying nanoparticles, garnering much attention.

In order to correctly and accurately apply machine learning methods for nanoparticle shape analysis, particle segmentation in the pre-processing step is critical in identifying and extracting nanoparticle metrology information. There are numerous methods for particle segmentation, ranging from threshold-based to more sophisticated deep-learning-based approaches.

Threshold-based image segmentation methods, including global and local thresholding methods such as Otsu’s method [22], Sauvola algorithm [23], and Niblack algorithm [24], are widely utilized. Some threshold-based methods such as cross-entropy thresholding [25] and variational theory-based methods [26] possess good adaptive capabilities, enabling automatic determination of the optimal separation threshold. Despite their prevalence, these methods, which depend on single or limited thresholds for segmenting images, frequently encounter difficulties in processing complex TEM images. Such images often present challenges like uneven background intensities [27,28] and variable particle intensities [29], hindering the achievement of optimal segmentation results.

To overcome the shortcomings of threshold-based approaches, machine-learning-based image segmentation approaches have gained much interest. Unsupervised machine learning segmentation approaches, such as k-means [30,31,32], mean shift [33,34,35,36,37], and GrabCut [38,39,40], show significant improvement over threshold-based methods. A recent report has demonstrated the effectiveness of combined template matching and image segmentation in extracting contours of platinum and core–shell nanoparticle catalysts from a complex background arising from the catalyst support [41]. However, this method may miss particles with shapes that deviate from the “template” or those with a high-contrast background.

On the other hand, supervised, deep learning methods such as convolutional neural networks (CNNs) can achieve very accurate particle segmentation even with highly complex background structures. However, deep learning methods require large amount of experimental or simulated training data. In the case of TEM images of nanoparticles, considering the varieties of imaging modalities and particle background features (such as support in the case of catalysis or cell features in nanoparticle uptake in cells), such deep learning approaches are undesirable as they require a priori training data sets, making automation for a wide range of TEM nanoparticle images very challenging.

Herein, we have developed a new method for nanoparticle segmentation using Shannon-entropy-optimized slope difference distribution (SEO-SDD). This method is an unsupervised clustering method capable of automatically finding the optimum image intensity clusters, thereby achieving particle segmentation from images. Such a method overcomes the shortcomings of intensity-threshold-based methods that generally fail when there are large background intensity variations. Moreover, as this method is unsupervised, it does not require a priori data training needed for the deep learning methods.

The effectiveness of our method is demonstrated on a deliberately chosen exemplar nanoparticle system of detonation nanodiamond (DND). DNDs are ≈5 nm diamond nanoparticles that are synthesized by the detonation process [42]. Due to their small particle size, flexible surfaces for functionalization, and biocompatibility, they have shown great promise for biomedical applications in drug delivery [43,44,45,46], gene therapy [47,48,49], tissue engineering, and bone surgery [50,51]. Because of their small size and weak contrast in TEM images, the segmentation of DNDs represents a challenging example for particle segmentation. Many existing automated particle segmentation processes for machine learning were demonstrated on nanoparticles with high image contrast, such as noble metal particles [52,53,54] and transition metal oxides [41], making them easier to identify due to stronger contrast differences from the background.

Moreover, we chose to use cryo-TEM images of frozen DND dispersion, which give noisier images compared to conventional TEM images. In addition, TEM images of DNDs taken up by cells were chosen as another example, as the complex features within the cells represent another great challenge in DND particle segmentation. The successful application of these DND examples demonstrate that our methods are efficient, effective, and robust.

## 2. Methodology

Generally, the workflow of machine learning (ML) morphology categorization for nanoparticles (Figure S1) is divided into two parts: the first part is pre-processing, which converts the raw image data into nanoparticle contour datasets for the subsequent ML algorithm. This part involves the denoising of raw images, followed by segmentation of nanoparticles. The second part is the application of the ML algorithm for shape categorization. As mentioned in the introduction, the focus of this work is on particle segmentation. In this section, we firstly describe our new Shannon-entropy-optimized slope difference distribution (SEO-SDD) particle segmentation method. Then, the ML algorithm, the sample preparation, and image acquisitions will be described.

### 2.1. Particle Segmentation: Automated Shannon-Entropy-Optimized Slope Difference Distribution (SEO-SDD) Method

**Figure 1:**
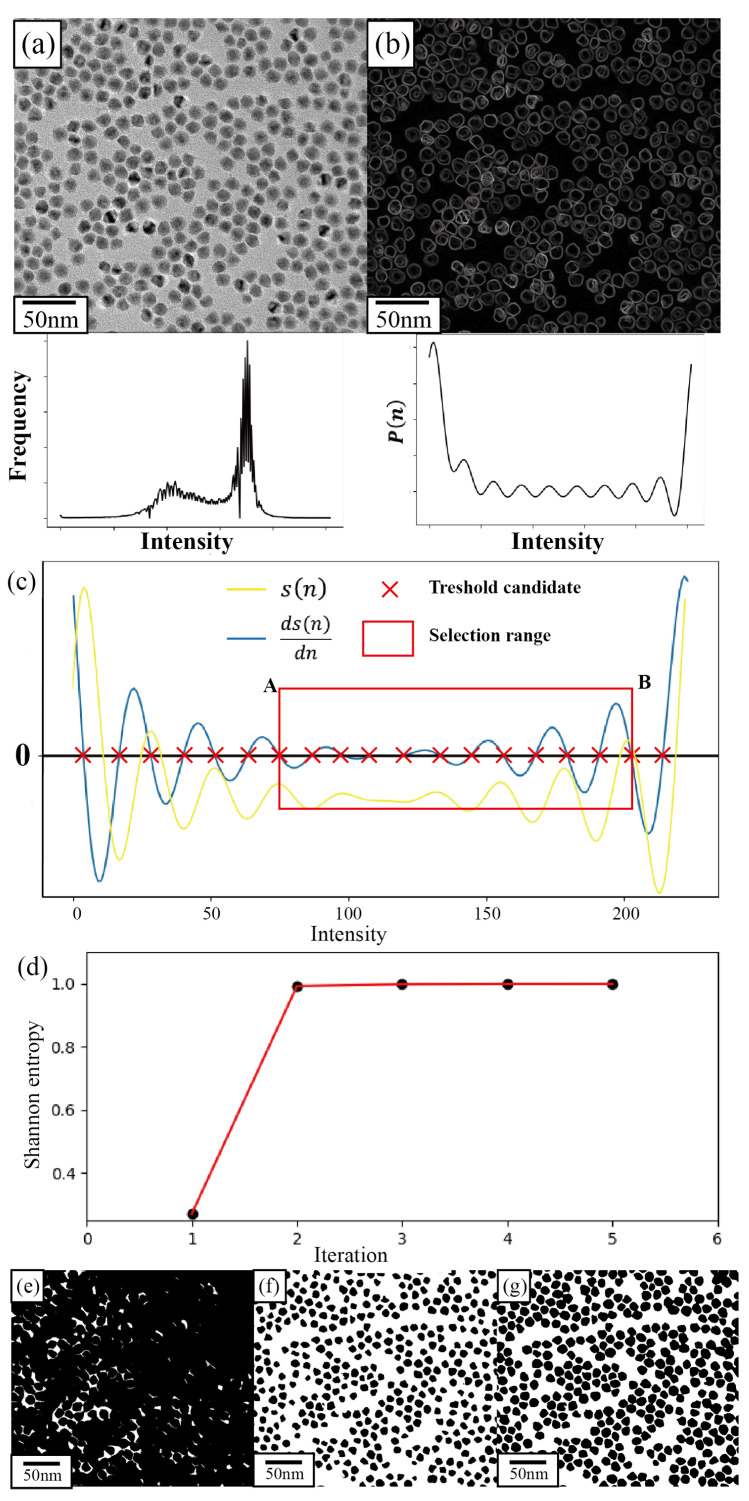
Process of Shannon-entropy-optimized slope difference distribution (SEO-SDD) method for nanoparticle segmentation from TEM images. (**a**) Raw TEM image with its corresponding intensity histogram; (**b**) gradient image with the corresponding normalized gradient histogram; (**c**) process of threshold range selection. The yellow curve (s(n)) represents the distribution of slope differences obtained based on the gradient histogram, while the blue curve ($\frac{ds(n)}{dn}$) represents the differential curve of the slope difference distribution. Red crosses denote candidate threshold values, and the red rectangle indicates the selection range for the optimal thresholds. A and B are respectively the upper and lower limits of the threshold selection range; (**d**) plot of the Shannon entropy values calculated in the iterative optimization process; (**e**–**g**) segmentation result in the 1st, 3rd, and 5th iteration, giving the Shannon entropy values of 0.30282, 0.99924, and 0.99999, respectively.

The first step of the pre-processing is denoising. Here, we employed a non-local mean (NLM) denoising algorithm [55] to reduce noise levels. Subsequently, we applied an SEO-SDD particle segmentation method (Figure 1) to obtain an accurate binary image of the nanoparticle.

As TEM images can exhibit various types of noise [56] arising from sample preparations, electron beam configuration, electron doses, and the detectors [57,58,59], some TEM images of nanoparticles can be particularly noisy. Examples include electron-beam-sensitive materials that require imaging at a low-electron dose, low-atomic-number materials such as carbon-based nanoparticles, and cryo-TEM images of frozen biological and nanoparticle dispersion samples [60]. These factors contribute to a low signal-to-noise ratio in the images, resulting in a significant amount of noise and low particle contrasts.

We chose NLM denoising as it is an effective approach for noise reduction and has been demonstrated to work well in electron microscopy images and spectroscopy maps [61,62]. The key principle of NLM is to find similar regions within the image and replace the original pixel values with the weighted mean pixel values of these regions, aiming to achieve denoising.

After applying NLM denoising, we obtain a higher signal-to-noise ratio TEM image, as shown in Figure 1a. Next, we use a fully automated Shannon-entropy-optimized slope difference distribution (SEO-SDD) method that we have developed to extract the particles in the image and convert it into a binary image (Figure 1g).

For a TEM image, the intensity of the image generally changes rapidly near the edges of particles. This means that there is an intensity gradient near the edges. Such a feature is common for TEM images (and other types of microscopy images) and is independent of the background intensity, contrast, and intensity variation within the particles. The principle of the SDD method utilizes such a feature to remove the background, thereby identifying the particles.

The SDD method, firstly developed by Wang [63], is a method for determining the center of data clusters in unsupervised machine learning (a more detailed description of the SDD method is given in Supplementary Materials Section I). It has been applied in fluorescence and scanning electron microscopy images [64]. It has primarily been used for particle and cell counting rather than for automated particle contour extraction. The workflow of the SEO-SDD method is shown in Figure 1, and the formulation of the SDD method is described below.

For a given image (Figure 1a), I(x), its corresponding gradient image (Figure 1b), $I_{g}\left(x\right)$, can be computed by combining the magnitude and the direction of the gradient image

(1) $$\begin{array}{cc} \left|I_{g}\left(x\right)\right| & =\sqrt{I_{i}^{2}\left(x\right)+I_{j}^{2}\left(x\right)} \end{array}$$

(2) $$\begin{array}{cc} \Theta & =atan2\left(I_{j}\left(x\right),I_{i}\left(x\right)\right) \end{array}$$

where $I_{i}$ denotes the gradient along x-coordinates and $I_{j}$ along y-coordinates. The fundamental principle of the SDD method is to find the extrema of the slope difference distribution function, s(x), from the gradient image, $I_{g}\left(x\right)$. The normalized gradient image intensity histogram distribution, P(n), is defined as

(3) $$P\left(n\right)=\frac{f_{n}}{f_{max}},$$

where $f_{n}$ denotes the frequency of a pixel intensity value *n* and $f_{max}$ is the maximum frequency of a pixel value. For a given point *n* in the histogram distribution P(n), two slopes—one on the left, $a_{L}\left(n\right)$, and the other on the right, $a_{R}\left(n\right)$—of the point adjacent to *n* can be calculated. The slope difference distribution function, s(n), is then defined as

(4) $$s\;\left(n\right)=a_{L}\;\left(n\right)-a_{R}\;\left(n\right).$$

The segmentation of particles in an image can then be treated as finding the centers of different clusters from s(n) by setting the derivative of s(n) to zero, given as

(5) $$\frac{ds\left(n\right)}{dn}=0;\;n\left(x\right)>0$$

By sorting and selecting the intersections of the derivative with the horizontal axis, we can then obtain a set of values as the centers of the “intensity clusters” (marked as red crosses in Figure 1c), which are the candidate intensity thresholds for P(n). Therefore, this method provides a more flexible approach for nanoparticle segmentation compared to conventional histogram-threshold-based approaches [21,65,66,67]

Having obtained a set of candidate threshold values based on Equation (5), the next challenge of the SDD method is to select the optimum thresholds. Previously, such a parameter requires manual input and can suffer from overly harsh background intensity removal [65,67] or excessive remaining noise. In order to achieve fully automated and robust particle segmentation, we propose a Shannon-entropy-optimized [68] binary search algorithm to determine the optimum intensity thresholds. It should be noted that the conventional binary search algorithm searches for the value sequentially when the value being sought is known [69]. In our case, since the optimal threshold is not known, a figure-of-merit evaluation function is needed to determine the optimum thresholds.

Here, we chose Shannon entropy [68], *H*, as the evaluation criterion. Shannon entropy is used to quantify the complexity or uncertainty of the data. For an image with a pixel intensity value, *n*, its Shannon entropy can be defined as

(6) $$H=-\Sigma_{i}p\left(n_{i}\right)\log_{2}p\left(n_{i}\right)$$

where $p(n_{i})$ is the probability that the pixel value takes on a specific intensity value $n_{i}$. The total entropy for the image therefore is given as

(7) $$H\left(N\right)=H_{Nanoparticles}+H_{Background}$$

The larger the value of the entropy, the more information an image contains. In other words, the data have lower entropy when an image contains more similar information. Therefore, by maximizing H(N), it is possible to achieve automated and robust particle segmentation.

The steps of the Shanon entropy optimization in our SEO-SDD method are described below:

1. Set the initial range of threshold selection by choosing the appropriate upper, *B*, and lower, *A*, limits. The range can be read from the curve of the differential of slope difference (shown in Figure 1c). The upper limit *B* differentiates the background from particles, and the lower limit *A* differentiates particles with different intensities.
2. Calculate the median and its corresponding Shannon entropy. The essence of the binary search algorithm lies in iteratively narrowing the search range by comparing the median value, mid=(A+B)/2, with the currently considered optimal threshold. As the actual optimal value remains to be identified, we compute the Shannon entropy of the median, H(mid), at each iteration and compare it with the entropy from the previous iteration.
3. Narrow the search range by comparing H(mid) and H(mid+1). If the entropy obtained using the current threshold mid is higher than mid+1, then adjust the search range to between *A* and mid. Otherwise, adjust the search range to between mid+1 and *B*.
4. Reaching the maximum *H* when A=B. The threshold value that maximizes H(N) is the best threshold for nanoparticle segmentation when *A* is equal to *B*.

Figure 1d–g illustrate this iterative optimization process by plotting the Shannon entropy value of each iteration and the corresponding segmented images from the selective 1st, 3rd, and final iterations.

In order to demonstrate the effectiveness of the SEO-SDD method, we estimate the accuracies of particle counting with respect to the simulated images with a known number of particles, which can then be served as the ground truth, given as

(8) $$Accuracy=100\%-\frac{|counts_{SEO-SDD}-counts_{Groundtruth}|}{counts_{Groundtruth}}$$

However, for the experimental TEM images, the exact number of particles is to be determined. In these instances, we use expert manual counting as a benchmark for evaluating the segmentation results of the image. As manual counting inherently introduces biases, it is therefore not suitable to be used as “ground truth”. Instead, we calculate the relative error (RE) for each segmentation result with respect to the manual counting result. A smaller RE means smaller differences in particle counting between the segmentation method and manual counting, thereby indicating a greater accuracy of the result. RE here can be defined as

(9) $$RE=\frac{|counts_{segmentation}-counts_{Manual}|}{counts_{Manual}}\times 100\%$$

### 2.2. Unsupervised Machine Learning for Nanoparticle/Aggregate Shape Categorization

After particle segmentation, the edges of the particles (shapes) were identified by applying the Canny edge detection algorithm [70]. This is a widely used method due to its simplicity and robustness [71,72].

Having converted the TEM image data into the particle contour dataset, we then apply an unsupervised ML categorization algorithm that we have previously developed for the particle and aggregate shape categorization [21,67,73] (see Supplementary Materials Section II). As our method is an unsupervised machine learning algorithm (distinct from, e.g., “deep learning” methods), it does not require a priori simulated and experimental training datasets and is therefore generally applicable to any nanoparticle/aggregate materials.

In brief, we apply the hierarchical agglomerative clustering method with the average linkage [67] to categorize the parameterized particle shapes. This clustering method builds a hierarchy of clusters and therefore does not require pre-determined optimum numbers of shape clusters. Thus, the hierarchical clustering method allows for full automation. Finally, the optimum numbers of clusters are determined automatically by applying internal cluster validity indexes (CVIs), with the optimum numbers being the common local extrema of each CVI.

It is worth mentioning that, in the case of aggregates of DNDs, as the attributes for the aggregates are different from the isolated particles, we have developed a different clustering method to categorize the aggregates [73]. Briefly, this method firstly identifies the aggregates by dividing the pre-processed image into grid cells. The choice of the grid cell is optimized to achieve high accuracy in shape categorization by testing a pre-determined range of grid-cell sizes. The grid-cell intensity histograms were initially categorized into three groups (kn) to differentiate the image background (k1), aggregate edges (k2), and aggregate interiors (k3), using the hierarchical agglomerative clustering method with the average linkage. Next, in order to distinguish the morphology of different aggregates, we need to perform a second categorization to include the first and second order nearest neighbors of a given grid cell. The optimum number of clusters of 3 was determined, which were termed as clusters, ropes, and chains. Clusters are defined as aggregates larger than three DNDs in diameter, ropes smaller than three DNDs in width, and chains approximately equal to one DND in width. More details can be seen in Supplementary Materials Section II C.

### 2.3. Sample Preparation and TEM Imaging

DND dispersions were prepared via a freeze-plunging method for cryo-TEM imaging. Approximately 1 wt% of each sample in deionized water was sonicated for ca. 30 min first and then 4.5 µL of the sample was deposited as a droplet onto the glow-discharged grid (R2/2 Quantifoil copper grids, Jena, Germany) using a Leica grid plunger.

The TEM samples of quantum dot nanoparticles were prepared by drop-casting nanoparticle dispersions onto the holey-carbon-film-coated Cu TEM grids (results in Supplementary Materials Section IV).

For the HeLa cell uptake of DNDs, DNDs were firstly ingested by HeLa cells using the standard process described in detail in the Supplementary Information. For preparing the TEM sample, the cells were postfixed in 1% OsO4 in 0.1 mol Na cacodylate buffer using a BioWave Pro + Microwave Tissue Processor (Ted Pella, Inc., Redding, CA, USA), washed again in Milli Q water, dehydrated with a graded series of ethanol, infiltrated with resin (ProSciTech Pty Ltd., Kirwan, QLD, Australia), and polymerized at 60 °C overnight. Ultrathin sections (70 nm) were collected onto carbon-coated copper TEM grids, which were then post stained with uranyl acetate (2%) and lead citrate (2%). Afterward, images were acquired using a JEOL TEM-1400 (Tokyo, Japan) operating at 120 keV.

The quantum dots were imaged using JEOL F200 (Tokyo, Japan), operated at 200 kV, using the bright-field TEM (BF-TEM) mode. The frozen DND dispersions were imaged using a Talos Arctica TEM (Thermo Fisher Scientific, Waltham, MA, USA) with an acceleration voltage of 200 keV.

## 3. Results and Discussion

In this section, we firstly validate the effectiveness of the SEO-SDD method by using a simulated nanoparticle image. We then test the method on two challenging types of TEM images: highly noisy cryo-TEM images of frozen DND dispersion in water and BF-TEM images of DND uptake in HeLa cells. We deliberately chose DND particles as an example due to the challenges that they present in terms of small particle size and lower image contrast (compared to the typically demonstrated noble metal particles), as mentioned in the introduction.

Having demonstrated the effectiveness of the SEO-SDD method, we then show how the results can be applied for accurate particles and aggregate shape categorization using unsupervised machine learning methods that we have reported previously [21,67,73] on the examples of quantum dot particles (see Supplementary Materials Section IV) and, again, DND aggregates.

### 3.1. Effectiveness of SEO-SDD Method

#### 3.1.1. Simulated Image

**Figure 2:**
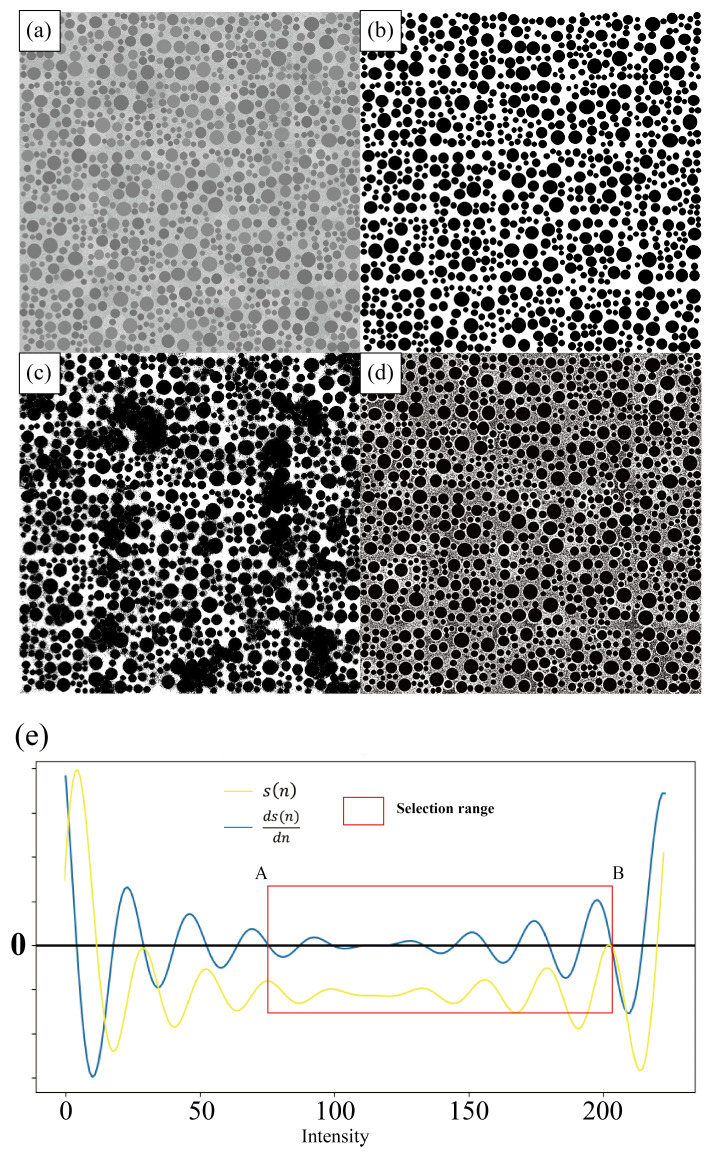
Comparison of SEO-SDD segmentation and other thresholding-based segmentation methods on a simulated image including 1028 particles with uneven background. (**a**) Simulated image; (**b**) SEO-SDD segmentation result; (**c**) histogram thresholding result; (**d**) dynamic thresholding result; (**e**) process of threshold range selection. The yellow curve (s(n)) represents the distribution of slope differences obtained based on the gradient histogram, while the blue curve ($\frac{ds(n)}{dn}$) represents the differential curve of the slope difference distribution. Red rectangle indicates the selection range for the optimal thresholds. A and B are respectively the upper and lower limits of the threshold selection range.

Figure 2a presents a simulated image containing 1,028 circular-shaped nanoparticles, each distinct in size and intensity levels. The background intensity of this image is intentionally uneven, incorporating several regions of sharp-intensity transitions. Figure 2e shows the distribution of slope differences based on the gradient histogram (yellow curve) and the differential curve of the slope difference distribution ($\frac{ds(n)}{dn}$, blue curve). The zeros of the $\frac{ds(n)}{dn}$ are candidate threshold values and the red rectangle indicates the selection range for the optimal thresholds. Figure 2b–d display the segmentation results using the SEO-SDD method, histogram thresholding, and dynamic thresholding, respectively. It can be observed that histogram thresholding fails to adequately mitigate the effects of the uneven background, thereby falsely identifying nanoparticles in these regions. Although dynamic thresholding marginally diminishes the impact of the uneven background by accentuating the contrast between the background and the particles, it largely leaves the background unaltered in the segmentation output. In the SEO-SDD results, despite few instances where closely spaced particles overlap, the particle outlines are predominantly extracted, with a good accuracy of 97%. The accuracy, determined using Equation (8), is listed in Table 1. The findings demonstrate that SEO-SDD achieves high accuracy. Subsequently, this methodology was applied to the experimental TEM images.

#### 3.1.2. Experimental TEM Images of DND Aggregates

**Figure 3:**
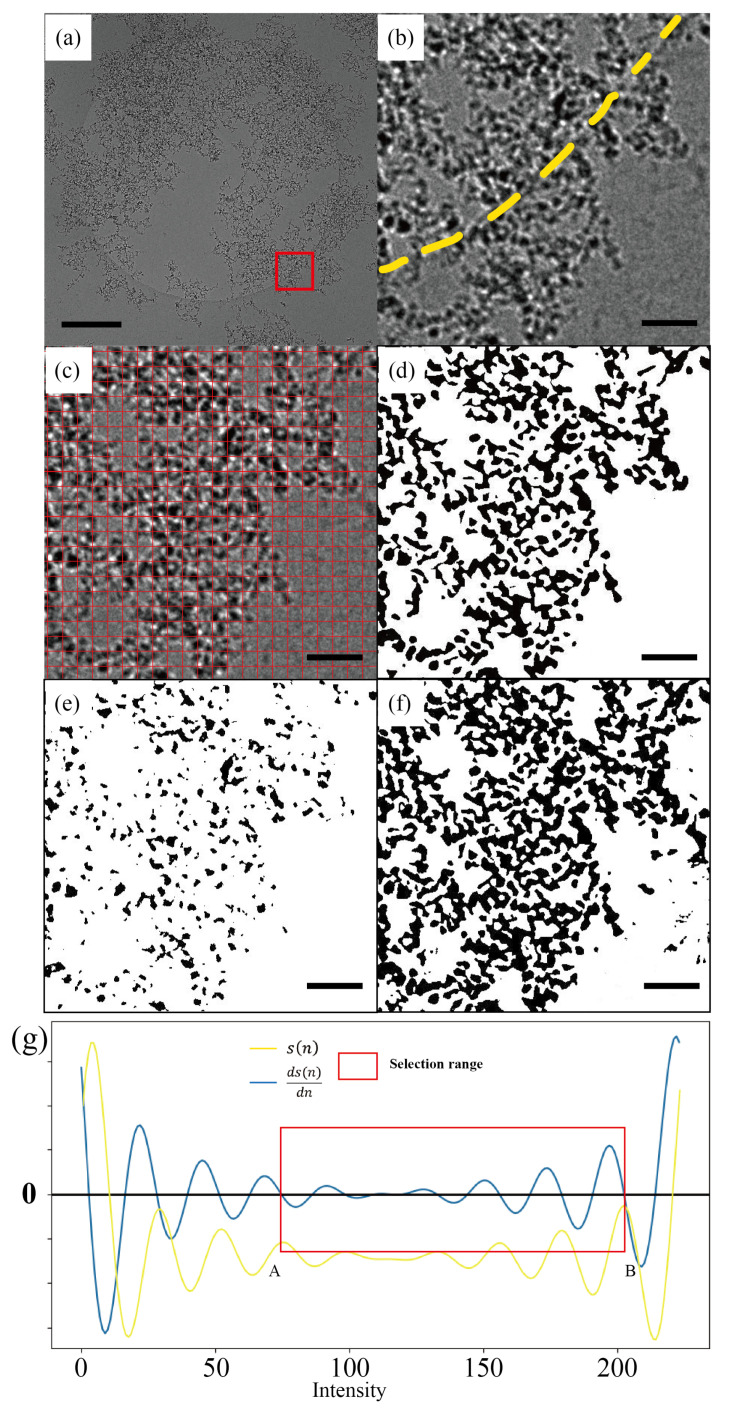
Comparisons of SEO-SDD segmentation and other thresholding-based segmentation methods on a cryo-TEM image of DND suspension supported on a “quanti-foil” amorphous carbon film. (**a**) Raw image; (**b**) magnification of the red area in (**a**). The boundary of hole and carbon film are indicated by yellow dashed lines; (**b**,**c**) covered with red grid lines for manual counting; (**d**) SEO-SDD segmentation result; (**e**) histogram thresholding result; (**f**) dynamic thresholding result; (**g**) process of threshold range selection. The yellow curve (s(n)) represents the distribution of slope differences obtained based on the gradient histogram, while the blue curve ($\frac{ds(n)}{dn}$) represents the differential curve of the slope difference distribution. Red rectangle indicates the selection range for the optimal thresholds. A and B are respectively the upper and lower limits of the threshold selection range. The scale bar in (**a**) is 500 nm, and those in (**b**–**f**) are all 50 nm.

Figure 3a shows a cryo-TEM image of frozen DND dispersion on a “quantifoil” amorphous carbon film. It can be seen that the DNDs form aggregates in water, with polydispersity and complex aggregate morphologies composed of elongated rope-like and cluster-shaped aggregates, as reported previously [74]. It has been reported that rope aggregates promote better drug biomolecule absorption and can be beneficial for cellular uptake through penetration of the membranes [75]. The cluster-shaped aggregates, on the other hand, can be utilized as nanoporous structures for slower and controlled drug release [76].

In this test experimental TEM image given in Figure 3a, its center shows a distinct ring-shaped uneven background area, which is a circular hole in the “quant foil film”, supporting the DND dispersion. Figure 3b shows an enlargement of the red square region in Figure 3a, highlighting the different background intensity levels between the hole and the carbon film. The segmentation result using the SEO-SDD method is given in Figure 3d in comparison with two other threshold-based methods.

It can be seen that, in the process of removing the uneven background, the histogram threshold method (Figure 3e) loses many nanoparticles as well as sacrifices the particle contours. On the other hand, dynamic thresholding (Figure 3f) appears to deal with such a background better, though it nonetheless misses many pores within the DND aggregates that were successfully identified by the SEO-SDD method (Figure 3d).

In order to quantify the number of nanoparticles in the images, we referred to the machine-learning-based method specifically designed for the classification of DND aggregates [73], briefly mentioned in Section 2.2 and detailed in Supplementary Materials Section II C. The key aspect of counting DND particles when they are aggregates is to utilize the “grid cells” applied to the image. The selection of grid cell size is optimized and is close to the size of DND particles. Thus, by categorizing the intensity histograms within each grid cell (details in Supplementary Materials Section II C), we can then deduce the numbers of DND particles. The manual counting of DND particles was conducted in a similar fashion. The “grid cells” were applied to the image as shown in Figure 3c, and then the DND particles were manually counted by counting the grid cells with intensities covering more than 50% of its area. The relative differences (REs) can then be directly compared among different segmentation methods with respect to the raw image. To reduce the errors inherited in manual counting, we employed the average of multiple manual counts as the reference for comparison.

The inaccuracies in particle segmentation from the threshold-based methods are reflected by means of particle counting relative differences, listed in Table 2. It shows that the SEO-SDD method is most accurate, the dynamic thresholding slightly under-filtering with similar performance, and the histogram thresholding grossly over-filtering.

**Figure 4:**
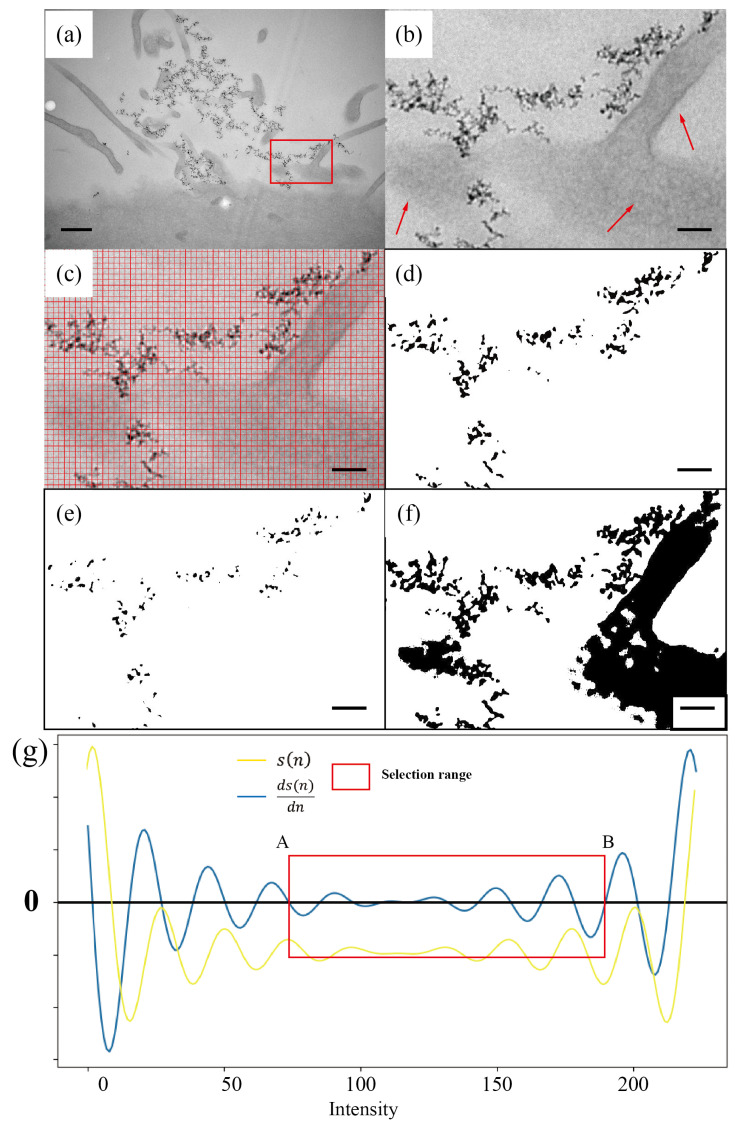
Comparison of SEO-SDD segmentation and other thresholding-based segmentation methods on a BF-TEM image of DNDs taken up by HeLa cells. (**a**) Raw image; (**b**) magnification of the red area in (**a**). Cellular structure is indicated by red arrows; (**b**,**c**) covered with red gridlines for manual counting; (**d**) SEO-SDD segmentation result; (**e**) histogram thresholding result; (**f**) dynamic thresholding result; (**g**) process of threshold range selection. The yellow curve (s(n)) represents the distribution of slope differences obtained based on the gradient histogram, while the blue curve ($\frac{ds(n)}{dn}$) represents the differential curve of the slope difference distribution. Red rectangle indicates the selection range for the optimal thresholds. A and B are respectively the upper and lower limits of the threshold selection range. The scale bar in (**a**) is 500 nm, and those in (**b**–**f**) are all 100 nm.

Figure 4 shows the second example of DNDs uptake by HeLa cells. The BF-TEM image of a slice of negatively stained HeLa cells, given in Figure 4a, demonstrates the highly uneven contrast arising from the complex features of cells as well as the darker contrast from the DND aggregates. As shown in Figure 4b, the red arrows point to the synapse of the cell. Due to the staining, the features of cells have relatively stronger contrast compared to typical TEM images of nanoparticles supported on thin amorphous carbon films. This imposes a greater challenge in segmenting nanoparticles from the background.

It can be seen that, even with complex and irregular cell features as the “background” present in the image, our SEO-SDD method can still successfully segment DND particles (Figure 4d) with very good accuracy, as listed in Table 3. The histogram thresholding method (Figure 4e), while achieving the removal of uneven backgrounds from the cell features by over-filtering, unfortunately sacrifices the correct segmentation of DNDs. Therefore, its RE is relatively high, exceeding 59%.

On the other hand, dynamic thresholding (Figure 4f) experiences difficulties in filtering the background in this case. It incorrectly identified almost all cellular structures in the image as nanoparticles. The RE presented in Table 3 exceeds 790%, indicating that dynamic thresholding in this case is highly unreliable.

### 3.2. Particle and Aggregate Shape Categorization through Unsupervised Machine Learning

Having demonstrated the effectiveness and robustness of our SEO-SDD particle segmentation method, in this section, we then apply this method in the pre-processing for particle/aggregate contour extraction for the unsupervised machine learning that we have reported previously [21,67,73]. Again, we have chosen DNDs dispersion and cell uptake as examples here. As these two examples are DND aggregates, we have shown the ML analysis of the nanoparticle system of quantum dots in the Supplementary Information Section IV for a better flow of the manuscript.

### 3.3. Cryo-TEM Image of DND Aggregates in PBS

**Figure 5:**
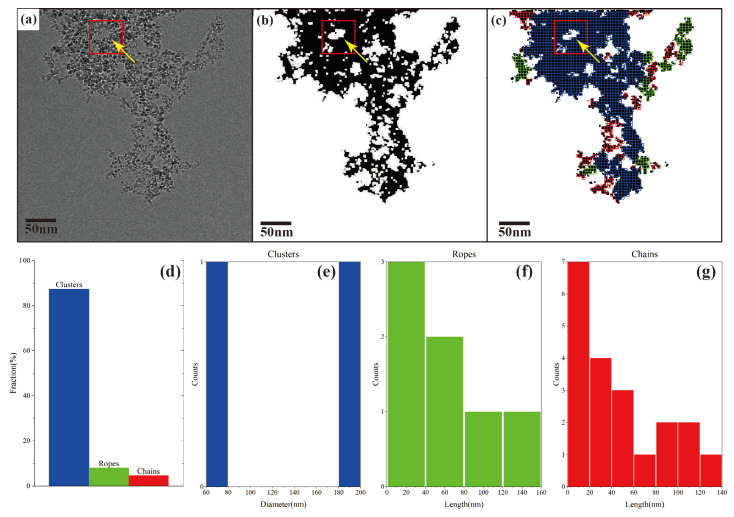
ML analysis of DND aggregate morphology categorization from a cryo-TEM image of DNDs in PBS. The small pore within DND particles in (**a**–**c**) is indicated by red box and yellow arrow. (**a**) Raw cryo-TEM image with the yellow arrow indicating a pore among the aggregates; (**b**) SEO-SDD segmented binary image; (**c**) aggregate morphology categorized groups overlay on (**b**), with cluster shape group shown in blue, rope shape group in green, and chain shape group in red; (**d**) morphology fraction of the three aggregate morphology groups; (**e**) diameter distribution of the cluster-shaped aggregates; (**f**) length distribution of rope-shaped aggregates; and (**g**) length distribution of chain-shaped aggregates.

For the use of DNDs in biomedical applications, their control of the aggregation behavior is critical to their performance. In order to understand the DNDs aggregation state in biomedical-relevant media, here, we show that the SEO-SDD segmentation method can successfully identify and extract DND aggregates for ML morphology categorization. Figure 5a shows a cryo-TEM image of DND dispersion in phosphate buffered saline (PBS), which is a common medium for cell cultures. It can be seen that the image is noisy and the background appears to be “textured”. This is due to the presence of salt in the solution. Under cryogenic conditions, these ions are likely to precipitate from the solution and form small crystals. These crystals produce a strong scattering effect, thereby reducing the clarity and contrast of the image. This is precisely because there are a large number of ions in PBS adsorbed on DNDs, therefore promoting the aggregation of DNDs. As a result, most of the DNDs in the image exist in the form of large aggregates, with small pores (an example of the pore is marked by the yellow arrow in Figure 5a) among the DND particles. This can result in small-contrast differences between the particles and the pores (background), giving difficulties in particle/aggregate segmentation.

Figure 5b shows the DND aggregate segmentation using the SEO-SDD method. We can see that, even in areas where DNDs are highly aggregated, SEO-SDD can still accurately extract the contours. The small pores between the aggregates are also accurately determined. Based on the segmented image given in Figure 5b, the aggregate morphologies were then categorized using our ML method [73]. In this approach, the results of image segmentation undergo erosion and dilation processing before being aligned with three distinct types of aggregate frameworks: clusters, ropes, and chains. Subsequently, the classification outcome depicted in Figure 5c is achieved. This method is described in Section II C of the Supplementary Information. The aggregates were divided into three morphology groups, labelled in blue, green, and red colors. Their corresponding morphology fractions (in terms of areal concentration) and size distributions are plotted in Figure 5d–g, respectively. The aggregate morphology analysis shows that the most of the aggregates, not surprisingly, are cluster-shaped aggregates (blue). The other two types of aggregate shapes, which we termed “ropes” (green) and “chains” (red), have lower fractions and exhibit relatively short lengths (mostly < 60 nm). Such a result is expected as a high-salt-concentration environment can cause nanoparticles, including DNDs, to form dense aggregates.

### 3.4. DND Uptake in HeLa Cells

For the use of nanoparticles for any biomedical application, studies of the cell uptake of nanoparticles are important in assessing the viabilities before any further development. Moreover, the shape of particles/aggregates has been established to be an important factor for the uptake [7]. Hence, here, we again use the DNDs as an example to show that our SEO-SDD segmentation method is a robust pre-processing method for the analysis of aggregate morphology distribution even in the cells.

**Figure 6:**
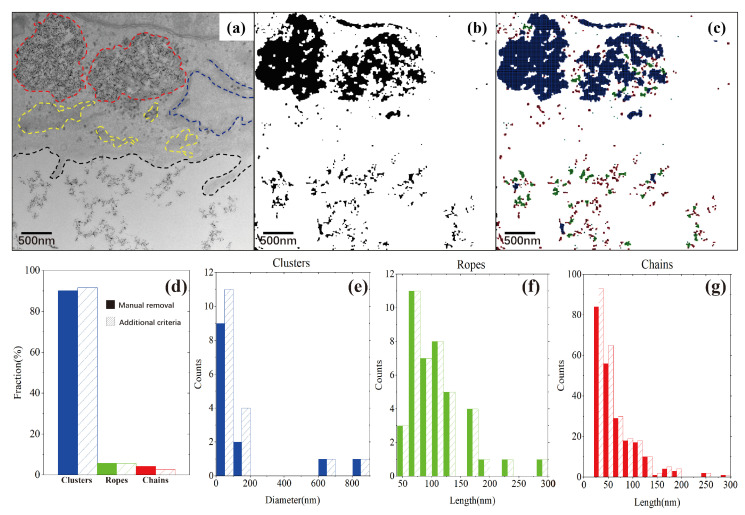
ML analysis of DND aggregate morphology categorization from a BF-TEM image of DNDs uptake in HeLa cells. (**a**) Raw BF-TEM image with the colored dotted lines indicating the cell internal features; (**b**) SEO-SDD segmented binary image; (**c**) aggregate morphology categorized groups overlayed onto (**b**); (**d**) fraction of the three aggregate morphology groups, with the manually segmented results shown in solid bars and the SEO-SDD results in shaded bars; (**e**) diameter distribution of the cluster-shaped aggregates; (**f**) length distribution of rope-shaped aggregates; and (**g**) length distribution of chain-shaped aggregates.

Figure 6a shows the BF-TEM image of the chemically fixed and negatively stained HeLa cell with DNDs ingested after 12 h. The DNDs can be seen as present both inside and outside the cell. The resultant DND segmentation is given in Figure 6b. It can be seen that even the background intensity has large variations, most of the background can still be removed equally using the SEO-SDD method. Moreover, the cell structure contrasts, including synapses, microvesicles, cell membranes, and endosomes, marked by a dashed line in Figure 6a, are successfully removed. However, we note that small fractions of cellular structures in the upper left corner and upper center of the image are not correctly removed. This is primarily due to the high contrast of the membrane structures in the cell. In order to further remove the large cell features, we have applied an additional size convexity constraint in the SEO-SDD process. Figure 6c shows the DND aggregate shape categorization analysis with the three major shape groups color-coded and overlayed onto the segmented image using our ML method. To demonstrate the accuracy of our SEO-SDD pre-processing and the ML shape analysis, Figure 6d–g compare the morphology distributions and aggregate size histograms using manual (solid bars) and SEO-SDD segmentation (shaded bars).

Based on the quantitative morphological analysis from the ML method, we find that the outcome of SEO-SDD processing is very close to the ground truth obtained through manual processing, although some fractions of the cell structures are falsely identified as DNDs. Our result shows that the SEO-SDD method can achieve a 96.33% accuracy when processing images containing such complex cellular structures.

## 4. Conclusions

In this work, we have developed an efficient, robust, and highly automated nanoparticle/aggregate segmentation method from TEM images. The Shannon-entropy-optimized slope difference distribution method, based on image intensity gradient and automatic optimum threshold selection, has been demonstrated to be successful on TEM images with a highly complex background. The SEO-SDD method is more efficient compared to other existing segmentation methods as it does not require a priori training datasets, which are critical for deep-learning-based segmentation methods.

The effectiveness of our method is demonstrated on (cryo-)TEM images of very small (5 nm) detonation nanodiamond particle dispersion and uptake in cells. Such examples represent great challenges in terms of small particle size and relatively low particle contrasts with a noisy and complex background. Our results show that the SEO-SDD method that we have developed provides a highly automated and accurate analysis of size and shape distributions of nanoparticles/aggregates. Moreover, even for TEM images with nanoparticles in cells, our method is sufficiently accurate and robust for extracting and correctly categorizing their shape distributions.

The ability to statistically analyze the size and shape distributions of nanoparticle/aggregate dispersion and cell uptake from (cryo-)TEM images will certainly make a significant contribution to advancing the understanding and applications of nanoparticles for biomedical applications. Moreover, while our methodology was only demonstrated on detonation nanodiamonds and quantum dot nanoparticles, it should be directly applicable to other nanoparticle systems for nanomedicine and biomedical applications, such as liposomes and polymeric micelles.

## Figures and Tables

**Table 1:** Comparisons of particle counting accuracies based on particle segmentation from the simulated image using the SEO-SDD, dynamic and histogram thresholding methods.

Methods	Number of Particles	Accuracy (%)
Ground truth	1028	100
SEO-SDD	997	96.98
Dynamic threshold	915	89.00
Histogram threshold	740	71.98

**Table 2:** Comparisons of the DND particle counting relative differences (with respect to manual counting) based on particle segmentation from the cryo-TEM image of DND dispersion using SEO-SDD, dynamic, and histogram thresholding methods.

Methods	Number	RE (%)
Manual counting	20,362	0
SEO-SDD	19,934	2.10
Dynamic threshold	20,906	2.67
Histogram threshold	12,360	39.30

**Table 3:** Comparisons of the DND particle counting relative differences, REs, (with respect to manual counting) from the BF-TEM image of HeLa cell uptaking DNDs, processed using SEO-SDD, dynamic, and histogram thresholding methods.

Methods	Number	RE (%)
Manual counting	2932	0
SEO-SDD	2816	3.96
Dynamic threshold	26,170	792.56
Histogram threshold	1191	59.38

## Data Availability

Data are contained within the article and Supplementary Materials.

## Supplementary Materials

The following are available online at https://www.mdpi.com/article/10.3390/nano14141169/s1. In the supplementary materials, we offer comprehensive descriptions of the original SDD method, the ML algorithms used for analyzing the shapes of nanoparticles and aggregates, and the techniques for acquiring TEM images referenced in the main text. Additionally, we provide a concise overview of the Python code employed to implement our approach. Refs. [77,78,79,80,81,82,83,84,85,86] are cited in Supplementary Materials.

## References

[1] Raffa V., Vittorio O., Riggio C., Cuschieri A. (2010). Progress in nanotechnology for healthcare. Minim. Invasive Ther. Allied Technol..

[2] Feng H.P., Tang L., Zeng G.m., Zhou Y., Deng Y.C., Ren X., Song B., Liang C., Wei M.y., Yu J.F. (2019). Core-shell nanomaterials: Applications in energy storage and conversion. Adv. Colloid Interface Sci..

[3] Xie C., Niu Z., Kim D., Li M., Yang P. (2019). Surface and interface control in nanoparticle catalysis. Chem. Rev..

[4] Li Y., Liu Q., Shen W. (2011). Morphology-dependent nanocatalysis: Metal particles. Dalton Trans..

[5] Khot L.R., Sankaran S., Maja J.M., Ehsani R., Schuster E.W. (2012). Applications of nanomaterials in agricultural production and crop protection: A review. Crop. Prot..

[6] Das S., Sen B., Debnath N. (2015). Recent trends in nanomaterials applications in environmental monitoring and remediation. Environ. Sci. Pollut. Res..

[7] Wang W., Gaus K., Tilley R.D., Gooding J.J. (2019). The impact of nanoparticle shape on cellular internalisation and transport: What do the different analysis methods tell us?. Mater. Horizons.

[8] Garcia-Alvarez R., Hadjidemetriou M., Sanchez-Iglesias A., Liz-Marzan L.M., Kostarelos K. (2018). In vivo formation of protein corona on gold nanoparticles. The effect of their size and shape. Nanoscale.

[9] Carnovale C., Bryant G., Shukla R., Bansal V. (2019). Identifying Trends in Gold Nanoparticle Toxicity and Uptake: Size, Shape, Capping Ligand, and Biological Corona. ACS Omega.

[10] Yang W., Wang L., Mettenbrink E.M., DeAngelis P.L., Wilhelm S. (2021). Nanoparticle Toxicology. Annu. Rev..

[11] Han X., Xu K., Taratula O., Farsad K. (2019). Applications of Nanoparticles in Biomedical Imaging. Nanoscale.

[12] McNamara K., Tofail S.A.M. (2017). Nanoparticles in biomedical applications. Adv. Phys. X.

[13] Suri S.S., Fenniri H., Singh B. (2007). Nanotechnology-based drug delivery systems. J. Occup. Med. Toxicol..

[14] Danaei M., Dehghankhold M., Ataei S., Hasanzadeh Davarani F., Javanmard R., Dokhani A., Khorasani S., Mozafari M.R. (2018). Impact of Particle Size and Polydispersity Index on the Clinical Applications of Lipidic Nanocarrier Systems. Pharmaceutics.

[15] Wu Z., Yang S., Wu W. (2016). Shape control of inorganic nanoparticles from solution. Nanoscale.

[16] Grzelczak M., Pérez-Juste J., Mulvaney P., Liz-Marzán L.M. (2008). Shape control in gold nanoparticle synthesis. Chem. Soc. Rev..

[17] An K., Somorjai G.A. (2012). Size and shape control of metal nanoparticles for reaction selectivity in catalysis. ChemCatChem.

[18] Chang L.Y., Cervera Gontard L., Dunin-Borkowski R.E., Ozkaya D. (2010). Resolving the Structure of Active Sites on Platinum Catalytic Nanoparticles. Nano Lett..

[19] Cervera Gontard L., Chang L.Y., Hetherington C.J.D., Kirkland A.I., Ozkaya D., Dunin-Borkowski R.E. (2007). Aberration-Corrected Imaging of Active Sites on Industrial Catalyst Nanoparticles. Angew. Chem. Int. Ed..

[20] Ealia S.A.M., Saravanakumar M.P. (2017). A review on the classification, characterisation, synthesis of nanoparticles and their application. Proceedings of the IOP Conference Series: Materials Science and Engineering.

[21] Wen H., Xu X., Cheong S., Lo S.C., Chen J.H., Chang S.L., Dwyer C. (2021). Metrology of convex-shaped nanoparticles via soft classification machine learning of TEM images. Nanoscale Adv..

[22] Bangare S.L., Dubal A., Bangare P.S., Patil S. (2015). Reviewing Otsu’s method for image thresholding. Int. J. Appl. Eng. Res..

[23] Senthilkumaran N., Vaithegi S. (2016). Image segmentation by using thresholding techniques for medical images. Comput. Sci. Eng. Int. J..

[24] Senthilkumaran N., Kirubakaran C. (2014). Efficient implementation of Niblack thresholding for MRI brain image segmentation. Int. J. Comput. Sci. Inf. Technol..

[25] Brink A.D., Pendock N.E. (1996). Minimum cross-entropy threshold selection. Pattern Recognit..

[26] Chan F.H., Lam F.K., Zhu H. (1998). Adaptive thresholding by variational method. IEEE Trans. Image Process..

[27] Pu S., Gong C., Robertson A.W. (2020). Liquid cell transmission electron microscopy and its applications. R. Soc. Open Sci..

[28] Liu K.L., Wu C.C., Huang Y.J., Peng H.L., Chang H.Y., Chang P., Hsu L., Yew T.R. (2008). Novel microchip for in situ TEM imaging of living organisms and bio-reactions in aqueous conditions. Lab Chip.

[29] Srnová-Šloufová I., Lednický F., Gemperle A., Gemperlová J. (2000). Core—Shell (Ag) Au bimetallic nanoparticles: Analysis of transmission electron microscopy images. Langmuir.

[30] Krishna K., Murty M.N. (1999). Genetic K-means algorithm. IEEE Trans. Syst. Man, Cybern. Part B.

[31] Likas A., Vlassis N., Verbeek J.J. (2003). The global k-means clustering algorithm. Pattern Recognit..

[32] Na S., Xumin L., Yong G. Research on k-means clustering algorithm: An improved k-means clustering algorithm. Proceedings of the 2010 Third International Symposium on Intelligent Information Technology and Security Informatics.

[33] Comaniciu D., Meer P. Mean shift analysis and applications. Proceedings of the Seventh IEEE International Conference on Computer Vision.

[34] Comaniciu D., Meer P. (2002). Mean shift: A robust approach toward feature space analysis. IEEE Trans. Pattern Anal. Mach. Intell..

[35] Derpanis K.G. (2005). Mean shift clustering. Lecture Notes.

[36] Carreira-Perpinán M.A. (2015). A review of mean-shift algorithms for clustering. arXiv.

[37] Wu K.L., Yang M.S. (2007). Mean shift-based clustering. Pattern Recognit..

[38] Xu N., Price B., Cohen S., Yang J., Huang T. (2017). Deep grabcut for object selection. arXiv.

[39] Han S., Tao W., Wang D., Tai X.C., Wu X. (2009). Image segmentation based on GrabCut framework integrating multiscale nonlinear structure tensor. IEEE Trans. Image Process..

[40] Wang Z., Lv Y., Wu R., Zhang Y. (2023). Review of GrabCut in Image Processing. Mathematics.

[41] Noval J.J.S., Gómez-Merchán R., Leñero-Bardallo J.A., Gontard L.C. (2023). TEMAS: A Flexible Non-AI Algorithm for Metrology of Single-Core and Core-Shell Nanoparticles from TEM Images (Part. Part. Syst. Charact. 2/2023). Part. Part. Syst. Charact..

[42] Krüger A., Kataoka F., Ozawa M., Fujino T., Suzuki Y., Aleksenskii A.E., Vul’ A.Y., Ōsawa E. (2005). Unusually tight aggregation in detonation nanodiamond: Identification and disintegration. Carbon.

[43] Ho D., Wang C.H.K., Chow E.K.H. (2015). Nanodiamonds: The intersection of nanotechnology, drug development, and personalized medicine. Sci. Adv..

[44] Chauhan S., Jain N., Nagaich U. (2020). Nanodiamonds with powerful ability for drug delivery and biomedical applications: Recent updates on in vivo study and patents. J. Pharm. Anal..

[45] Giammarco J., Mochalin V.N., Haeckel J., Gogotsi Y. (2016). The adsorption of tetracycline and vancomycin onto nanodiamond with controlled release. J. Colloid Interface Sci..

[46] Benson V., Amini A. (2020). Why nanodiamond carriers manage to overcome drug resistance in cancer. Cancer Drug Resist..

[47] Bertrand J.R., Pioche-Durieu C., Ayala J., Petit T., Girard H.A., Malvy C.P., Le Cam E., Treussart F., Arnault J.C. (2015). Plasma hydrogenated cationic detonation nanodiamonds efficiently deliver to human cells in culture functional siRNA targeting the Ewing sarcoma junction oncogene. Biomaterials.

[48] Leung H.M., Chan M.S., Liu L.S., Wong S.W., Lo T.W., Lau C.H., Tin C., Lo P.K. (2018). Dual-Function, Cationic, Peptide-Coated Nanodiamond Systems: Facilitating Nuclear-Targeting Delivery for Enhanced Gene Therapy Applications. ACS Sustain. Chem. Eng..

[49] Zhang X.Q., Chen M., Lam R., Xu X., Ōsawa E., Ho D. (2009). Polymer-Functionalized Nanodiamond Platforms as Vehicles for Gene Delivery. ACS Nano.

[50] Fox K., Ratwatte R., Booth M.A., Tran H.M., Tran P.A. (2020). High Nanodiamond Content-PCL Composite for Tissue Engineering Scaffolds. Nanomaterials.

[51] Nunes-Pereira J., Silva A., Ribeiro C., Carabineiro S., Buijnsters J., Lanceros-Méndez S. (2017). Nanodiamonds/poly(vinylidene fluoride) composites for tissue engineering applications. Compos. Part B Eng..

[52] Wang X., Li J., Ha H.D., Dahl J.C., Ondry J.C., Moreno-Hernandez I., Head-Gordon T., Alivisatos A.P. (2021). AutoDetect-mNP: An unsupervised machine learning algorithm for automated analysis of transmission electron microscope images of metal nanoparticles. JACS Au.

[53] Liang Z., Nie Z., An A., Gong J., Wang X. (2019). A particle shape extraction and evaluation method using a deep convolutional neural network and digital image processing. Powder Technol..

[54] Lee B., Yoon S., Lee J.W., Kim Y., Chang J., Yun J., Ro J.C., Lee J.S., Lee J.H. (2020). Statistical Characterization of the Morphologies of Nanoparticles through Machine Learning Based Electron Microscopy Image Analysis. ACS Nano.

[55] Buades A., Coll B., Morel J.M. (2011). Non-local means denoising. Image Process. Line.

[56] Kushwaha H.S., Tanwar S., Rathore K., Srivastava S. De-noising filters for TEM (transmission electron microscopy) image of nanomaterials. Proceedings of the 2012 Second International Conference on Advanced Computing & Communication Technologies.

[57] Russo F. (2003). A method for estimation and filtering of Gaussian noise in images. IEEE Trans. Instrum. Meas..

[58] Azzeh J., Zahran B., Alqadi Z. (2018). Salt and pepper noise: Effects and removal. JOIV Int. J. Inform. Vis..

[59] Boyat A.K., Joshi B.K. (2015). A review paper: Noise models in digital image processing. arXiv.

[60] Danino D. (2012). Cryo-TEM of soft molecular assemblies. Curr. Opin. Colloid Interface Sci..

[61] Mevenkamp N., Yankovich A.B., Voyles P.M., Berkels B. Non-local Means for Scanning Transmission Electron Microscopy Images and Poisson Noise based on Adaptive Periodic Similarity Search and Patch Regularization. Proceedings of the VMV.

[62] Mevenkamp N., MacArthur K.E., Tileli V., Ebert P., Allen L.J., Berkels B., Duchamp M. (2020). Multi-modal and multi-scale non-local means method to analyze spectroscopic datasets. Ultramicroscopy.

[63] Wang Z. (2017). Determining the clustering centers by slope difference distribution. IEEE Access.

[64] Wang Z. (2016). A new approach for segmentation and quantification of cells or nanoparticles. IEEE Trans. Ind. Inform..

[65] Kapur J.N., Sahoo P.K., Wong A.K. (1985). A new method for gray-level picture thresholding using the entropy of the histogram. Comput. Vision, Graph. Image Process..

[66] Davies E.R. (1990). Machine Vision: Theory, Algorithms, Practicalities (Signal Processing and its Applications).

[67] Wen H., Luna-Romera J.M., Riquelme J.C., Dwyer C., Chang S.L. (2021). Statistically representative metrology of nanoparticles via unsupervised machine learning of TEM Images. Nanomaterials.

[68] Shannon C.E. (1948). A mathematical theory of communication. Bell Syst. Tech. J..

[69] Williams L.F. A modification to the half-interval search (binary search) method. Proceedings of the 14th Annual Southeast Regional Conference.

[70] Canny J. (1986). A computational approach to edge detection. IEEE Trans. Pattern Anal. Mach. Intell..

[71] Sharifi M., Fathy M., Mahmoudi M.T. A classified and comparative study of edge detection algorithms. Proceedings of the International Conference on Information Technology: Coding and Computing.

[72] Kalbasi M., Nikmehr H. (2020). Noise-Robust, Reconfigurable Canny Edge Detection and its Hardware Realization. IEEE Access.

[73] Kuschnerus I.C., Wen H., Ruan J., Zeng X., Su C.J., Jeng U.S., Opletal G., Barnard A.S., Liu M., Nishikawa M. (2023). Complex Dispersion of Detonation Nanodiamond Revealed by Machine Learning Assisted Cryo-TEM and Coarse-Grained Molecular Dynamics Simulations. ACS Nanosci. Au.

[74] Chang S.L., Reineck P., Williams D., Bryant G., Opletal G., El-Demrdash S.A., Chiu P.L., Ōsawa E., Barnard A.S., Dwyer C. (2020). Dynamic self-assembly of detonation nanodiamond in water. Nanoscale.

[75] Schrand A.M., Hens S.A.C., Shenderova O.A. (2009). Nanodiamond particles: Properties and perspectives for bioapplications. Crit. Rev. Solid State Mater. Sci..

[76] Ho D. (2009). Nanomaterial-based therapy: A new generation of cancer treatment. Therapy.

[77] Wang D., Zhou S. Color image recognition method based on the prewitt operator. Proceedings of the 2008 International Conference on Computer Science and Software Engineering.

[78] Vairalkar M.K., Nimbhorkar S.U. (2012). Edge detection of images using sobel operator. Int. J. Emerg. Technol. Adv. Eng..

[79] Cherri A.K., Karim M.A. (1989). Optical symbolic substitution: Edge detection using prewitt, sobel, and roberts operators. Appl. Opt..

[80] Chaple G.N., Daruwala R.D., Gofane M.S. Comparisions of robert, prewitt, sobel operator based edge detection methods for real time uses on fpga. Proceedings of the 2015 International Conference on Technologies for Sustainable Development (ICTSD).

[81] Hu M.-K. (1962). Visual pattern recognition by moment invariants. IRE Trans. Information Theory.

[82] Arbelaitz O., Gurrutxaga I., Muguerza J., P´erez J.M., Perona I. (2013). An extensive comparative study of cluster validity indices. Pattern Recognit..

[83] Rousseeuw P.J. (1987). Silhouettes: A graphical aid to the interpretation and validation of cluster analysis. J. Comput. Appl. Math..

[84] Davies D.L., Bouldin D.W. (1979). A cluster separation measure. IEEE Trans. On Pattern Anal. Mach. Intell..

[85] Cali´nski T., Harabasz J. (1974). Communications in statistics—theory and methods. Commun. Stat..

[86] Pedregosa F., Varoquaux G., Gramfort A., Michel V., Thirion B., Grisel O., Blondel M., Prettenhofer P., Weiss R., Dubourg V. (2011). Scikit-learn: Machine learning in python. J. Mach. Learn. Res..

