# Supplementary material for: Efficientand Robust Automated Segmentation of Nanoparticles and Aggregates from Transmission Electron Microscopy Images with Highly Complex Backgrounds
Source: Nanomaterials (Basel). 2024 Jul 9;14(14):1169. doi: 10.3390/nano14141169 (PMC11279516; doi:10.3390/nano14141169)
Supplement: Supplementary file 1 [file nanomaterials-14-01169-s001.zip › nanomaterials-2911068-supplementary.pdf]

Supplementary Materials for "Efficient and Robust Automated Segmentation of Nanoparticles and Aggregates from TEM Images with Highly Complex Backgrounds"

Lishi Zhou, Haotian Wen, and Inga C. Kuschnerus

School of Materials Science and Engineering,

University of New South Wales, Sydney, Australia

Shery L.Y. Chang

Electron Microscope Unit, Mark Wainwright Analytical Centre,

University of New South Wales, Sydney, Australia and

School of Materials Science and Engineering,

University of New South Wales, Sydney, Australia

I. SLOPE DIFFERENCE DISTRIBUTION METHOD

The core of our work is to use shannon-entropy optimized slope difference distribution (SDD) to remove background from images. This part is mainly used to introduce the theory of background removal using the original SDD.

First, we use an operator to transform the denoised grayscale image into a gradient image. Prewitt operator [1], Sobel operator [2] and Roberts operator [3] are some frequently used operators which can detect the gradient of images. The Sobel operator has a small convolution kernel, which leads to low computational cost. It also considers the weights of neighboring pixels and exhibits good direction sensitivity [4]. Therefore, we choose to use the Sobel operator to detect the gradient of the image.

After obtaining the gradient image, it is common to encounter the issue of unclear edges in the resulting gradient image due to low contrast in some TEM images. To facilitate further processing, it is necessary to perform grayscale stretching on the image, which expands the grayscale range to its maximum extent (0-255). This helps to make the details in the image clearer.

Next, in order to distinguish between particle edges and the background in the image, we scan the pixel values of the entire image and obtain a normalized histogram distribution. The proportion of each pixel in the histogram P_i is calculated as follows:

$$P_i = \frac{N_i}{N_j} \tag{1}$$

$$i \in [1, 255], j \in [1, 255], j = argmaxN_j$$

where N_k means the number of appearance of pixels with a value of k and j is the maximum pixel value that appears in the image. It is important to note that peak normalization is used here, which further enhances the contrast of the image.

To make the normalized histogram smoother and eliminate some residual noise that may not have been removed by NLM, we need to apply filtering to the histogram. The approach is to apply the Discrete Fourier Transform (DFT) to the histogram to convert it to the frequency domain, and then only retain the low-frequency region and the highest-frequency region. The low-frequency region corresponds to the slowly varying parts in the grayscale image, such as the background and smooth areas. The high-frequency region corresponds

to the rapidly changing parts in the image, which are likely to be noise. After grayscale stretching, the regions with the maximum gradients in the image correspond to the edge contours of the particles, which should be retained as they represent the highest-frequency components.

Based on these ideas, we first apply DFT to the histogram function using the following approach:

$$F(K) = \sum_{x=1}^{255} P_i e^{-i\frac{2\pi kx}{255}} \tag{2}$$
$$K = 1, \cdots, 255$$

Next, we apply filtering to the function transformed into the frequency domain:

$$F'(K) = \begin{cases} F(K); K = 1, \cdots, K_1 \\ 0; K = K_1, \cdots, K_2 \\ F(K); K = K_2, \cdots, 255 \end{cases} \tag{3}$$

where $K_1, \cdots, K_2$ is the likely noisy part which should be filtered. In our research, after repeated trials, we found that setting the range as $K_1, \cdots, K_2 = 11, \cdots, 245$ achieves the optimum separation result. Then, we transform the processed normalized function back to the spatial domain:

$$P_i' = \frac{1}{T} \sum_{K=1}^{255} F'(K) e^{i\frac{2\pi kx}{255}} \tag{4}$$
$$x = 1, \cdots, 255$$

The filtered and smoothed histogram clearly represents the distribution of gradients in the image. By fitting these discrete points into a continuous function, we can identify the regions in the image where the peaks of the function occur, which indicate areas of concentrated gradient distribution. These regions likely correspond to the contours of particles or background areas. The valleys between each peak can be used as intensity thresholds to separate them. The discrete function can be fitted using the matrix form of the least squares method:

$$y_i = ax_i + b \tag{5}$$

$$[a, b]^T = (B^T B)^{-1} B^T Y \tag{6}$$

$$B = \begin{bmatrix} x_1 & 1 \\ x_2 & 1 \\ \vdots & \vdots \\ x_N & 1 \end{bmatrix} \tag{7}$$

$$Y = [y_1, y_2, \cdots, y_N]^T \tag{8}$$

Here, N represents the number of fitting points required to obtain the fitted function. The more points used for fitting, the more accurate the function becomes. However, using too many points may result in overfitting. In our research, we used the default number of fitting points $N = 15$. After obtaining the plot of the fitted function, to calculate the thresholds more accurately, it is necessary to compute the difference in slopes between the left and right adjacent points of each discrete point in the fitted function. This will stretch both the peak and valley values and make it easier to find the threshold.

After this process, we can obtain a set of values for candidate thresholds. These thresholds can be used to separate particles from the background as well as to separate different particles from each other. But as mentioned in the paper, these candidate values are often inaccurate when processing TEM images. This is our motivation to improve it.

II. UNSUPERVISED MACHINE LEARNING METHOD FOR PARTICLE AND AGGREGATE MORPHOLOGY CATEGORIZATION

The overall process of nanoparticle shape analysis based on machine learning (ML) methods is illustrated in Fig. S1. In the main text, we obtained the binary image as shown in Fig. S1 (c) using the SEO-SDD method. Next, an unsupervised machine learning method that we have developed previously was used for nanoparticles [5, 6] and aggregates [7] morphology distribution analysis. Here will introduce in detail the principles of the unsupervised machine learning method to categorize the morphology.

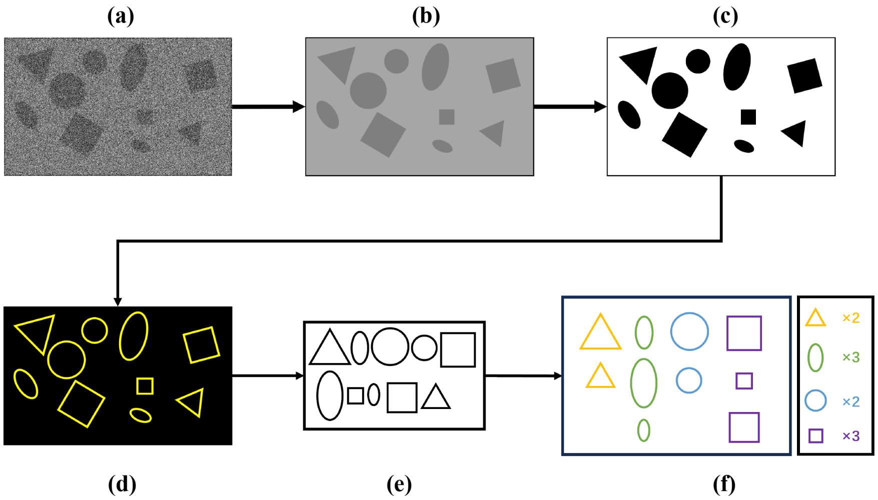

FIG. S1. Workflow of ML-based nanoparticle shape analysis. (a) raw image; (b) denoised image; (c) segmentation result; (d) edge detection result from (c); (e) shape extraction results by algorithm; (f) result of shape classification.

A. Shape descriptions for particles

For describing the shapes of nanoparticles, we chose Hu moments to parameterize the shape of all particles. Hu moment is a set of numbers (up to seven numbers) only related to particle shape, independent of particle direction, position, and size [8]. Each shape of nanoparticles could be converted into a Hu moment. If we regard this dataset as a point in a coordinate system with seven axes, all nanoparticles in the image can be distributed in this coordinate system as many different data points through different shapes. At this time, in this“Hu space” with seven coordinate axes, the distance between data points represents the shape similarity of the nanoparticles.

B. Hierarchical Clustering Algorithm

Once we obtain the shape descriptor of particles and aggregates, we then applied the hierarchical agglomerative clustering method with the average linkage to classify the parameterized particle shapes. Briefly speaking, a hierarchical clustering algorithm groups together data points (in our case, the particle contours) with similarities between them. This is achieved by using a measurement metric (in our case, the ”distance” between the pair of data points) and a linkage criteria, which specifies the similarity of data sets as a

function of pairwise distances of observations in the sets. This clustering method builds a hierarchy of clusters and therefore does not require a pre-determined optimum numbers of clusters. Use these possibilities of clustering, we can then apply the Cluster Validity Indexes (CVIs) [9] to determine the optimal number of clusters. To avoid bias towards a given set of a validation criteria, we choose three different CVIs: Silhouette [10], Davies-Bouldin [11] and Calinski-Harabaz [12]. These CVIs are selected from Scikit-learn machine learning library [13]. Thus, the hierarchical clustering method allows full automation.

C. Clustering algorithm for aggregate distribution

As the attributes for the aggregates associated with the properties are different from the isolated particles, we have developed a different clustering method to categorize the aggregates [7].

This method firstly identify the aggregates by dividing the pre-processed image into grid cells. The choice of the grid-cell is optimized to achieve high accuracy in shape categorization, by testing a pre-determined range of grid-cell sizes. The grid-cell intensity histograms were *initially* categorized into three groups (k_n) to differentiate the image background (k_1), aggregate edges (k_2), and aggregate interiors (k_3), using the hierarchical agglomerative clustering method with the average linkage. Next, in order to distinguish the morphology of different aggregates, we need to perform a second categorization to include the first and second order nearest neighbors of a given grid-cell. The optimum number of clusters of 3 was determined, which were termed: clusters, ropes and chains. Clusters are defined as aggregates larger than three DND in diameter, ropes smaller than three DND in width, and chains approximately equal to one DND in width. Finally, the analysis is completed by measuring the shape parameters of these aggregate outlines. The flowchart of this method and two examples applied in the main text are shown in Fig. S2.

In section 3.1 of the paper, We also referred this method for quantifying the number of nanoparticles in images. As mentioned above, when we convert aggregates in the image into grid cells, we no longer perform further classification. Instead we counted the number of grid cells occupying DND image intensities. Consequently, we can use the number of grid cells to replace the direct counting of nanoparticles (present in aggregate form and difficult to distinguish).

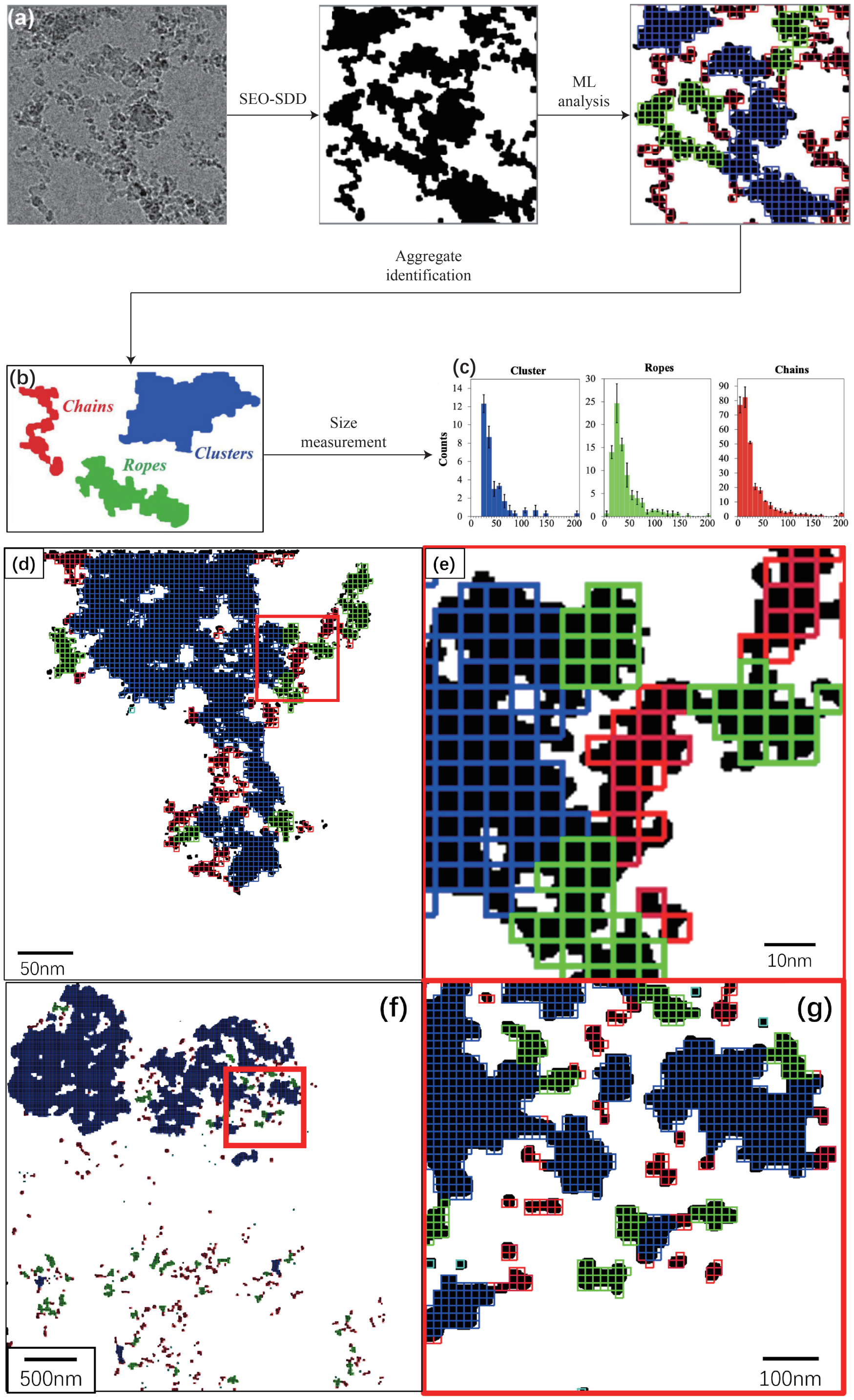

FIG. S2. The flow chart of ML analysis for aggregates. (a) Aggregates identification; (b) Aggregate morphohlogy categorization; (c) Aggregate size histogram. (d)-(e) Classification results of DNDs in PBS in the paper; (f)-(g) Classification results of DNDs uptake in HeLa cells in the paper.

III. ACQUISITION OF TEM IMAGES

In the paper we used many TEM images as examples, and this section details how these images were obtained.

A. Cryo-TEM images of DNDs

These DND suspensions were supplied by Daicel Corporation. These suspension samples are transformed from liquid to vitreous ice in a matter of microseconds using the freeze-plunging method. This method can keep the DNDs in its original state in the solution to facilitate the subsequent acquisition of cryo-TEM images. After each sample was sonicated in approximately 1 $wt\%$ ionized water for 30 minutes, 4.5 μL of the sample was dropped onto glow-discharged grid ($R2/2$ Quantfoil copper grids, Jena, Germany) using a Leica grid plunger at 25 °C and 89% humidity, with a blotting time of 3.5 seconds. Finally, images were acquired from the Talos Artica TEM (Thermo Fisher Scientific, Waltham, USA) with an accelerating voltage of 200 keV.

B. BF-TEM image of quantum dots

We used a BF-TEM image of quantum dots (QDs) in Fig. 5 in the paper. Samples of QDs were prepared by drop-casting nanoparticle dispersions onto the holey carbon film coated Cu TEM grids. Next, images were acquired using JEOL F200 (Akishima, Tokyo, Japan) in bright-TEM (BF-TEM) mode at an accelerating voltage of 200 keV.

C. BF-TEM images of DNDs in Hela cell

We used BF-TEM image of DNDs in Hela cell in Fig. 4, Fig. 7 and Fig. 8 in the paper. The acquisition of DNDs is similar to the previous description, so this section mainly introduces the culture process of HeLa cells. First, add 10% Fetal Bovine Serum (FBS) and 1% L-glutamine solution to the HeLa cell solution, place the above cell solution in dulbecco's modified eagle medium (DMEM) for culture, and then place the culture medium in a humidified incubator containing 5% carbon dioxide at 37 °C. During this process, the culture medium was changed every two days until the cells reached 80% confluency.

Once obtained cells that meet requirements, plate them onto a 12-well plate (10^5/well) containing a round sterile glass coverslip and let them grow for another 24 hours. In order to study the absorption of DNDs solutions of different concentrations by HeLa cells, a series of DNDs solutions with a minimum of $5\,\mu g\,mL^{-1}$ and a maximum of $50\,\mu g\,mL^{-1}$ were mixed with the above cells. Next, the cell culture medium was removed from the wells during the slow dropwise addition of 1 mL of fixative (room temperature) consisting of 2.5% glutaraldehyde in $0.2\,mol\,L^{-1}$ sodium cacodylate buffer. After completing the above steps, store the cells in a fridge at 4 °C overnight. During this process, the state of cells absorbing DNDs in solutions of different concentrations is fixed and preserved.

The following are sample preparation to obtain BF-TEM images of DNDs in HeLa cells. After removing the cell sample from the fridge, washed the cells with Milli Q®water. Then, postfixed them in 1% OsO_4 in $0.1\,mol\,L^{-1}$ Na cacodylate buffer using a BioWave Pro + Microwave Tissue Processor (Ted Pella, Inc., USA), washed again in Milli Q®water, dehydrated with a graded series of ethanol, infiltrated with resin (Procure, 812) and polymerised at 60 °C overnight. Ultrathin sections (70 nm) were collected onto carbon coated copper TEM grids, which were then post stained with uranyl acetate (2%) and lead citrate (2%). Afterwards, images were acquired using a JEOL TEM-1400 operating at 120 keV.

IV. QUANTUM DOTS PARTICLE SHAPE CATEGORIZATION USING UNSUPERVISED MACHINE LEARNING METHOD

The quantum dots (QD) nanoparticles shown in the TEM image are densely distributed with fairly strong background contrast from the amorphous carbon supporting film. After denoising by NLM, we use SEO-SDD to remove the background (Fig. S3 (c)) and convert it into a binary image. The segmented QDs were then analyzed by our ML method described in Section II A and B. The shape distribution of the QDs are represented by a 3D plots of the first three Hu moments (Fig. S3 (e)). In this three-dimensional space, the closer the data points are, the closer the nanoparticles they represent are similar in shape. The shape categorized distributions are shown in Fig. S3 (d) and (e) and listed in Table S1.

From the results, ML divided the particles in the image into 4 categories. In Fig. S3 (d), each class of different nanoparticles is labeled with a different color. The black nanoparticles were not included in the statistics because they were located at the edge of the image and

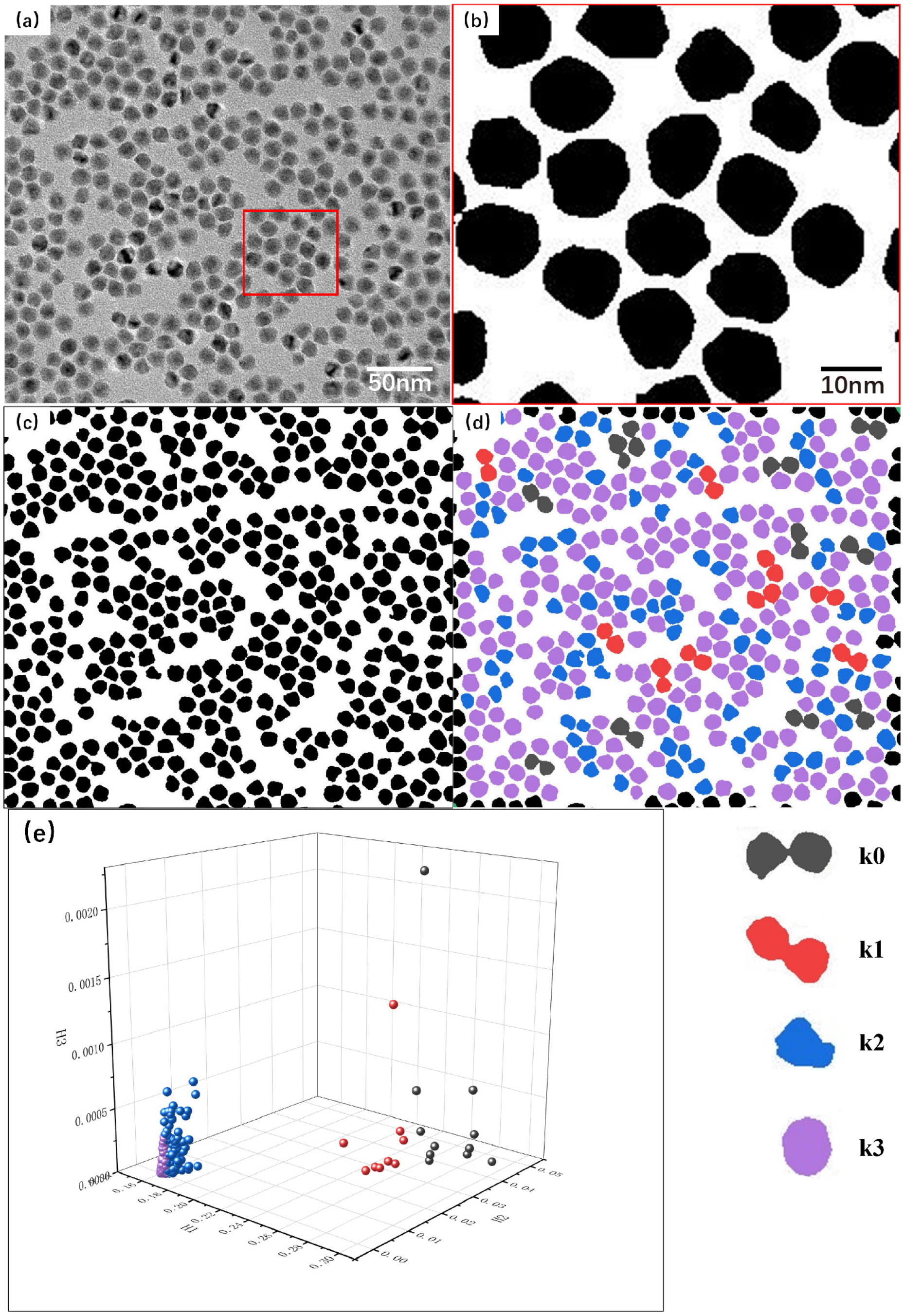

FIG. S3. Shape categorisation of QDs. (a) BF-TEM image of QDs; (b) zoom-in of the particle segmented image of the red square region shown in (a); (c) SEO-SDD particle segmented image; (d) shape categorization result; (e) QD shape distribution plotted as a function of the first three HU moments, with the shape groups color coded.

TABLE S1. Summary for the QDs shape analysis, where k denotes the shape group; Σ and Fraction are the number and the fraction of particles for each shape group; H1, H2 and H3 are the average of the first three Hu moments in each group.

k	Σ	Fraction	H1	H2($\times 10^{-2}$)	H3($\times 10^{-4}$)
0	11	3.24%	0.27 ± 0.01	3.93 ± 0.47	4.18 ± 6.49
1	9	2.65%	0.25 ± 0.01	2.93 ± 0.32	2.49 ± 4.14
2	92	27.06%	0.17 ± 0.00	0.14 ± 0.09	1.95 ± 1.68
3	228	37.06%	0.16 ± 0.00	0.04 ± 0.03	0.62 ± 0.56

their outlines were incomplete. We can find that among these five classes of particles, $k0$ and $k1$ are aggregates, $k2$ and $k3$ are separated nanoparticles. The 3D scatter diagram also confirmed our conjecture. Because they are separated particles, $k2$ and $k3$ are concentrated in the scatter diagram. Since the distribution rule of aggregate Hu moments is different from that of regular shapes, so the distribution of $k0$ and $k1$ in the scatter diagram is relatively scattered, and their location is far away from the separated nanoparticles. From Table S1, we can see that the aggregate in the image only account for a small part ($3.24\% + 2.65\% = 5.89\%$). The remaining separated particles also have two shapes, and their differences are mainly reflected in H2. If this image is analyzed manually, it is difficult to accurately classify the two particles into two categories. This once again illustrates the importance of ML for image analysis.

V. INTRODUCTION TO THE CODE FOR SEO-SDD

In the segmentation process of nanoparticles and aggregates, we utilized a Python-based program to execute our methodology. This section offers a comprehensive overview of the codes used in our analysis.

A. Overview of the code structure

Our approach employs a series of Python scripts, each dedicated to a distinct stage of the image processing pipeline which is corresponding with the methodology in main text.Below are the key steps in our script and the core Python functions used:

1. **Image Denoising**: The input TEM image is subjected to non-local means denoising via `cv2.fastNlMeansDenoisingColored` to reduce noise while preserving essential details, enhancing the image quality for further analysis.

2. **Edge Detection**: The Sobel operator, implemented through `cv2.Sobel`, processes the denoised image to compute gradients in both magnitude and direction. These gradients are crucial for delineating potential nanoparticle contours.

3. **Normalization and Histogram Analysis**: Post edge detection, the gradient image is normalized to improve contrast. Subsequently, a histogram analysis using `np.histogram` and `plt.plot` assesses the pixel value distribution, accentuating key features.

4. **Frequency Domain Filtering**: The histogram data is converted to the frequency domain using the Discrete Fourier Transform (`np.fft.fft`). A low-pass filter is then applied to refine the data, highlighting pertinent patterns.

5. **Slope Difference Calculation**: The filtered histogram undergoes analysis to detect slope differences, identifying local minima. This involves computing differences between adjacent slopes and applying peak finding algorithms (`scipy.signal.find_peaks`) to pinpoint potential nanoparticle boundaries.

6. **Entropy-Based Threshold Optimization**: The optimal threshold for image segmentation is identified by maximizing the combined information entropy of the foreground and background. This technique calculates entropy for multiple threshold values and employs a binary search to ascertain the optimal threshold, thereby achieving accurate segmentation.

7. **Thresholding**: We apply a binary threshold to the processed image using `PIL.Image.putpixel`. This isolates the contours of nanoparticles by removing pixels below a designated intensity value.

B. Discussion on code

The code employs a comprehensive and robust approach, spanning noise reduction to contour extraction. It provides adjustable parameters, allowing customization to suit various datasets and specific research requirements. The implementation of established techniques such as non-local means denoising, Sobel edge detection, and Discrete Fourier Transform enhances the reliability and robustness of image processing. Additionally, the integration of entropy-based threshold optimization improves precision in detecting nanoparticle boundaries, thereby increasing the code's overall accuracy and effectiveness. While versatile, certain computational stages, such as the DFT and entropy calculation, impose significant computational demands, necessitating adequate processing power for optimal execution, especially with larger or higher-resolution images.

Overall, our code is both concise and efficient, typically requiring no more than two minutes to process TEM images and generate binary image of contour for nanoparticles or aggregates in practical applications.

---

[1] Wang Dong and Zhou Shisheng. Color image recognition method based on the prewitt operator. In *2008 International Conference on Computer Science and Software Engineering*, volume 6, pages 170–173. IEEE, 2008.

[2] Manoj K Vairalkar and SU Nimbhorkar. Edge detection of images using sobel operator. *International Journal of Emerging Technology and Advanced Engineering*, 2(1):291–293, 2012.

[3] Abdallah K Cherri and Mohammad A Karim. Optical symbolic substitution: edge detection using prewitt, sobel, and roberts operators. *Applied optics*, 28(21):4644–4648, 1989.

[4] Girish N Chaple, RD Daruwala, and Manoj S Gofane. Comparisions of robert, prewitt, sobel operator based edge detection methods for real time uses on fpga. In *2015 International Conference on Technologies for Sustainable Development (ICTSD)*, pages 1–4. IEEE, 2015.

[5] Haotian Wen, José María Luna-Romera, José C Riquelme, Christian Dwyer, and Shery LY Chang. Statistically representative metrology of nanoparticles via unsupervised machine learn-

ing of tem images. *Nanomaterials*, 11(10):2706, 2021.

[6] Haotian Wen, Xiaoxue Xu, Soshan Cheong, Shen-Chuan Lo, Jung-Hsuan Chen, Shery LY Chang, and Christian Dwyer. Metrology of convex-shaped nanoparticles via soft classification machine learning of tem images. *Nanoscale Advances*, 3(24):6956–6964, 2021.

[7] Inga C Kuschnerus, Haotian Wen, Juanfang Ruan, Xinrui Zeng, Chun-Jen Su, U-Ser Jeng, George Opletal, Amanda S Barnard, Ming Liu, Masahiro Nishikawa, et al. Complex dispersion of detonation nanodiamond revealed by machine learning assisted cryo-tem and coarse-grained molecular dynamics simulations. *ACS Nanoscience Au*, 2023.

[8] Ming-Kuei Hu. Visual pattern recognition by moment invariants. *IRE transactions on information theory*, 8(2):179–187, 1962.

[9] Olatz Arbelaitz, Ibai Gurrutxaga, Javier Muguerza, Jesús M Pérez, and Iñigo Perona. An extensive comparative study of cluster validity indices. *Pattern recognition*, 46(1):243–256, 2013.

[10] Peter J Rousseeuw. Silhouettes: a graphical aid to the interpretation and validation of cluster analysis. *Journal of computational and applied mathematics*, 20:53–65, 1987.

[11] David L Davies and Donald W Bouldin. A cluster separation measure. *IEEE transactions on pattern analysis and machine intelligence*, (2):224–227, 1979.

[12] T Caliński and JJCiS-t Harabasz. Communications in statistics—theory and methods. *Communications in Statistics*, 3(1):1–27, 1974.

[13] Fabian Pedregosa, Gaël Varoquaux, Alexandre Gramfort, Vincent Michel, Bertrand Thirion, Olivier Grisel, Mathieu Blondel, Peter Prettenhofer, Ron Weiss, Vincent Dubourg, et al. Scikit-learn: Machine learning in python. *the Journal of machine Learning research*, 12:2825–2830, 2011.
